# Dietary complex probiotic supplementation changed the composition of intestinal short-chain fatty acids and improved the average daily gain of weaned piglets

**DOI:** 10.3389/fvets.2024.1424855

**Published:** 2024-06-21

**Authors:** Jianfei Zhao, Zhuoya Xie, Meiling Zheng, Wenjie Tang, Hui Diao, Heng Yin

**Affiliations:** ^1^School of Life Science and Engineering, Southwest University of Science and Technology, Mianyang, China; ^2^Sichuan Academy of Animal Science, Chengdu, China

**Keywords:** weaning weaned piglets, probiotics, intestinal morphology, short-chain fatty acids, growth performance

## Abstract

Probiotics are a group of active microorganisms that form colonies within the body and alter the composition of the flora in a specific area to provide benefits to the host. In this study, a total of 96 Duroc × Landrace × Yorkshire weaned piglets with an initial body weight (BW) of 8.56 ± 0.53 kg were employed in a randomized complete block design for a 28-day experiment. Pigs were randomly divided into two treatment groups: the control group (CON) and the complex probiotic group (CON + 0.2% probiotics), respectively. The study found that through the 28-day experiment, the average daily gain (ADG) of the complex probiotic group was significantly higher than that of the CON (*p* < 0.05). However, compared with the CON, the feed conversion efficiency significantly decreased on days 0–14 (*p* < 0.05). The addition of dietary complex probiotic significantly increased the villus height (VH) of duodenum and ileum, acetate, propionate, butyrate, and total short-chain fatty acids (SCFAs) in feces, and decreased fecal methyl mercaptans, acetic acid, and CO_2_ (*p* < 0.05). It concluded that feeding weaned piglets 0.2% complex probiotic increased the VH of duodenum and ileum, as well as changed the content of SCFAs in feces. This ultimately led to an increase in ADG.

## 1 Introduction

Weaning is a crucial stage in a pig’s life cycle. Stress during weaning can cause temporary or long-lasting damage to the intestinal barrier function, which will result in disease infections, anorexia, and economic losses in weaned piglets (1–3). In addition, weaning stress can reduce intestinal villus height (VH), which subsequently affects nutrient absorption (4). Therefore, it is important to alleviate weaning stress in piglets to minimize economic losses on farms.

Probiotics are a group of active microorganisms that form colonies within the body and alter the composition of the flora in a specific area to provide benefits to the host (5, 6). In recent years, probiotics have been extensively studied. The results showed that probiotics colonized the animal body and used nutrients in the intestinal tract that are difficult for the host to utilize (such as crude fiber) to carry out autotrophic metabolism and produce vitamins, amino acids, organic acids, enzymes, and other substances. This improves nutrient absorption by the animal, promotes intestinal health, and provides a certain degree of antimicrobial properties (2, 5–10). In addition, probiotics are considered an effective alternative to antibiotics, which have been partially banned due to the risk of antibiotic resistance (11). Therefore, adding probiotics to the diet is an effective way to alleviate weaning stress in piglets. Numerous previous studies have been conducted on the inclusion of probiotics in piglet diets (12–14). However, the efficacy of different probiotics to alleviate weaning stress requires further evaluation, particularly with the development of various types of probiotics. Previous studies have demonstrated that the addition of *Bacillus licheniformis*, *Bacillus subtilis*, *Lactobacillus acidophilus*, and *Saccharomyces cerevisiae* to the diet of weaned piglets has a significant positive impact on piglet growth performance (15–18). The resistance of different strains to the gastrointestinal environment varies. Furthermore, the effects of probiotics from different strains are multifactorial and strain-specific (19). Further investigation is required to ascertain whether different combinations of probiotics are beneficial or detrimental to piglets. The previous studies did not evaluate the addition of the mixture of four probiotics, namely *Bacillus licheniformis*, *Bacillus subtilis*, *Lactobacillus acidophilus*, and *Saccharomyces cerevisiae*, to piglets’ diet. Thus, this experiment aimed to evaluate the effects of the above probiotic mixture on growth performance, which could provide reference data for the use of different probiotics in piglets’ diet.

## 2 Materials and methods

### 2.1 Ethics statements and probiotics

The Institutional Review Board of the Southwest University of Science and Technology approved the animal study protocol (protocol code SM20220025). The probiotic product used in the study consisted of four types of mixed probiotics with an effective content of 5.1 × 10^7^ CFU/g *Bacillus licheniformis*, 6.3 × 10^7^ CFU/g *Bacillus subtilis*, 4.3 × 10^7^ CFU/g *Lactobacillus acidophilus*, and 2.5 × 10^7^ CFU/g *Saccharomyces cerevisiae*. The probiotics in this study were obtained from Sichuan Gao Fu Ji Biological Technology Co., Ltd. The amount of addition was 0.2% as recommended by the company. The production process adheres to production hygiene standards, and all probiotics that have undergone analysis have been found to be devoid of antibiotic resistant genes.

### 2.2 Experimental design, animals, and housing

A total of 96 4-week-old Duroc × Landrace × Yorkshire weaned piglets with an initial body weight (BW) of 8.56 ± 0.53 kg and weaned at 28 ± 1 day of age were employed in a randomized complete block design for a 28-day experiment. Pigs were randomly divided into two treatment groups based on BW and sex before the experiment. Each dietary treatment had 12 replicates with four pigs per replicate (two barrows and two gilts). The study involved two treatment groups: the control group (CON) and the probiotic group (CON + 0.2% probiotics). The diet used in CON (Table 1) was a compound feed in mash form, which was formulated to meet the nutrient requirements of weaned piglets (20). During the study, the pigs were housed in plastic floor pens (0.9 m × 1.6 m). They had unlimited access to feed and water via a self-feeder or nipple drinker. All pigs were disease free. The target room temperature of 20°C and humidity of 60% were maintained throughout the study.

**Table 1:** Ingredient and chemical composition of diets.

Ingredients	Content, %
Corn	61.20
Soybean meal (43%)	20.00
Fish meal	7.00
Whey powder	8.00
Soybean oil	0.35
CaHPO_4_	0.80
Limestone	0.50
Salt	0.20
Premix^1^	0.50
L-Lys-HCL (78.8%)	0.70
DL-methionine	0.45
L-Threonine	0.30
Total	100.00
Calculated nutrient level, %	
Digestible energy, MJ/kg	14.09
Crude protein	20.21
Total Ca	0.86
Total P	0.70
SID Lys	1.55
SID Met + Cys	0.90
SID Thr	0.92
Ca: P	1.22

^1^The premix provided the following per kg of diets: vitamin A 12,000 IU, vitamin D_3_ 2,500 IU, vitamin E 30 IU, vitamin K_3_ 3 mg, vitamin B_5_ 10 mg, vitamin B_12_ 27.6 mg, niacin 30 mg, choline chloride 400 mg, Mn (as MnO) 40 mg, Fe (as FeSO_4_·H_2_O) 90 mg, Zn (as ZnO) 100 mg, Cu (as CuSO_4_·5H_2_O) 8.8 mg, I (as KI) 0.35 mg, and Se (Na_2_SeO_3_) 0.3 mg. The proportion of corn was replaced with a 0.2% probiotic complex. SID, Standard ileal digestibility.

### 2.3 Fecal score and sample collection

The fecal consistency was evaluated twice a day at 08:00 and 20:00 in each pen by observing the feces discharged on the floor and a five-point scale (1: hard, dry pellets; 2: firm, formed stools; 3: soft, moist stools that retain shape; 4: soft, unformed stools that assume the shape of the container; and 5: watery liquid that can be poured) was used to score the feces. The fecal score values were averaged after summarizing the values on a weekly basis in circles. BW of each pig was measured on days 0, 14, and 28 to determine the average daily gain (ADG). Feed consumption was recorded daily by measuring the amount of feed fed and remaining in the pen to calculate the average daily feed intake (ADFI) and feed-to-gain ratio (F: G). Chromium oxide, an indigestible marker, was added to the diet at a rate of 0.5% for 4 days (during days 11–14 and days 25–28) before fecal collection by rectal massage on days 12–14 and 26–28 to calculate the digestibility values of dry matter (DM), crude protein (CP), and gross energy (GE). At 8 o’ clock on day 28, two pigs (one male and one female) were selected from each pen for jugular vein blood collection. The blood collection was placed in heparinized tubes for further analysis. After collecting, one pig per pen was randomly selected for euthanasia and weighed to ensure a 1:1 ratio of males to females within the treatment group. The patient was administered intramuscular ketamine and thiazide anesthesia, followed by intravenous pentobarbital sodium. The remaining pigs were individually weighed and their data were recorded. Following the execution, a midline incision was made to the abdomen and thorax. Duodenal specimens were collected 30 cm from the stomach, while jejunal and ileal specimens were collected 2 m and 30 cm before the ileocecal junction, respectively. The tissue samples were then rinsed in cold 0.9% saline and stored in a 10% formalin buffer to fix the villi and crypts. Additionally, samples from the end of the colonic contents (replacement of feces) weighing 20, 40, and 100 g were collected to assess the presence of microbiota, short-chain fatty acids (SCFAs), and noxious gases, respectively.

### 2.4 Nutrient digestibility and hematological profiles analyses

After drying in a forced-air oven and grinding through a 1 mm sieve, the dietary and fecal samples were analyzed. The method of Liu et al. (16) was used to determine the levels of calcium (Ca) and phosphorus (P) in the diet. The DM content of the feces was determined by drying them in a forced-air oven at 105°C for 2 h (21). The concentration of CP in the experimental diets and feces was determined by using the combustion method (21). Dietary and fecal GE were measured by using a fully automated calorimeter (BYLRY-3000 W). After nitrification, a spectrophotometer was used to measure the concentration of Cr_2_O_3_ in the diets and feces by reading the absorbance at 450 nm. The apparent total tract digestibility was determined by the levels of Cr, DM, CP, and GE in the feed and feces. Calculate the apparent total tract digestibility of the nutrient using the following equation ATTD = [1 − (Cr_d_/Cr_f_) × (N_f_/N_d_)] × 100% (Cr_d_ = Cr concentration in diets; Cr_f_ = Cr concentration in feaces; N_f_ = nutrient concentration in feaces; N_d_ = nutrient concentration in diets). Blood indicators were measured by using an automatic biochemical analyzer (BK-600, Biobased, Jinan City, Shandong Province, China) (21). Concentrations of interleukin-1β (IL-1β), interleukin-6 (IL-6), and interleukin-10 (IL-10) in weaned piglets were measured by using the ELISA kit from the Jiancheng Institute of Biological Technology in Nanjing, China. The cytokine analysis was conducted in duplicate following the manufacturer’s instructions. ELISA kits validated for weaned piglets (Nanjing Jiancheng Biology Co., Ltd.) were used to analyze the concentrations of IgA, IgG, and IgM, following the described methods.

### 2.5 Microbiota analysis

Fecal microbial assays were conducted by following the protocol described by Liu et al. (22). Total DNA from fecal samples was extracted by using Omega BioTec’s stool DNA isolation kit (Norcross, GA, United States). The experiment aimed to determine the concentrations of six bacterial species: *Prevotella*, *Bacteroides*, *Bifidobacterium*, *Escherichia coli*, *Lactobacillus*, and *Bacillus*. Table 2 displays the primer sequences and corresponding annealing temperatures. In the fluorescence-quantitative PCR reaction system, 11 μL were used, comprising of 2 μL of DNA, 2.7 μL of RNase-free water, 0.4 μL (10 M) of upstream and downstream primers, and 5.5 μL of 2 KAPA SYBR FAST qPCR Kit Master Mix. The reaction conditions were as follows: the temperature was set to 50°C for 2 min, followed by 95°C for 10 min. Then, 40 cycles of denaturation/annealing were carried out at 95°C for 15 s and 60°C for 1 min. Finally, a melting curve process was performed from 70 to 90°C, increasing by 0.5°C every 5 s. The fluorescence-quantitative reaction was performed on an ABI 7600 instrument.

**Table 2:** Primer sequence for microbiota.

Bacteria	Upstream primer (5′ → 3′)	Downstream primers (5′ → 3′)	AT, °C	R
*Prevotella*	CACCAAGGCGACGATCA	GGATAACGCCTGGACCT	60	(23)
*Bacteroides*	CGATGGATAGGGGTTCTGAGAGGA	GCTGGCACGGAGTTAGCCGA	60	(24)
*Bifidobacterium*	TCGCGTCYGGTGTGAAAG	GGTGTTCTTCCCGATATCTACA	60	(25)
*Escherichia coli*	CATGCCGCGTGTATGAAGAA	CGGGTAACGTCAATGAGCAAA	60	(26)
*Lactobacillus*	AGCAGTAGGGAATCTTCCA	ATTCCACCGCTACACATG	60	(27)
*Bacillus*	CCTACGGGAGGCAGCAGTAG	GCGTTGCTCCGTCAGACTTT	59	(28)

AT, Annealing temperature; R, Reference.

### 2.6 Determination of SCFAs

The homogenized powders obtained after lyophilization and pestling were extracted with 1 mL of methanol (gradient grade for liquid chromatography Li Chrosolv R Reag. Ph Eur EA). After 10 min of sonication, the samples were centrifuged at 6,000 rpm for 10 min, and the resulting supernatants were used for GC–MS analysis. The GC-MS analysis was performed by using a GC-MS-QP2010 Ultra with an autosampler (SHIMADZU) and the Rtx-wax capillary column (30 m, 0.25 mm i.d., 0.25 mm film thickness; SHIMADZU). The oven temperature was programmed to increase from 60 to 100°C at a rate of 5°C/min, with a 1 min hold; then to 150°C at a rate of 5°C/min, with a 5 min hold; and finally, to 225°C at a rate of 30°C/min, with a 20 min hold. A 2 mL of sample was injected at 230°C. Helium was used as the carrier gas with a flow rate of 1.2 mL/min. Electronic impact was recorded at 70 eV.

### 2.7 Gas emissions analyses

The fecal samples (100 g each) from each pen were placed in separate 2.6-L plastic containers with a small hole in the middle of one side. The containers were sealed with tape. The samples were allowed to ferment at room temperature (25°C) for 24 h. Then, approximately 2.0 cm above the fecal sample, 100 mL of headspace air was sampled. The small hole for gas detection was closed again after the gas was collected. Prior to measurement, the fecal samples were shaken manually for approximately 30 s. This was to break up any crusts on the surface and to homogenize the samples. A portable 6-in-1 gas detector (SGA-606, ingoan, Shenzhen, Guangdong Province, China) was used to measure the concentrations of NH_3_, H_2_S, mercaptan, acetic acid, and CO_2_.

### 2.8 Intestinal morphometry

The small intestine segments were cut into 5 mm sections and stained by using the HE method according to previous study (29). The VH and crypt depth (CD) were measured by randomly selecting 10 VH and 10 CD per section from images captured under a Carl Zeiss light microscope from each sample.

### 2.9 Statistical analyses

Data were analyzed by using the SAS (SAS Inst. Inc., Cary, NC, United States). After testing, all data are normally distributed. The *t*-test statistically analyzed the experimental data. Growth performance, nutrient digestibility, and fecal score were analyzed by using the pen as the experimental unit. Intestinal morphology, hematological profiles, fecal microbiota, fecal SCFAs, and fecal gas emissions were analyzed by using the individual pig as the experimental unit. Results with *p* < 0.05 were considered statistically significant.

## 3 Results

### 3.1 Growth performance, nutrient digestibility, and fecal score

There were significant changes in body weight (Day 14 and Day 28) and ADG (Day 0–28), and it was extremely significant (*p* < 0.01) on final body weight (Day 28) and ADG (Day 0–28) with the addition of dietary complex probiotics (Table 3; *p* < 0.05). F: G decreased when added complex probiotics to the diet on days 0–14 (*p* < 0.05). But compared with CON pigs, dietary complex probiotics supplementation did not affect ADFI (*p* > 0.05). Meanwhile, there was no effect on nutrient digestibility in weaned pigs after adding the complex probiotics to the diet, whether on day 14 or 28 (Table 4; *p* > 0.05). The addition of dietary complex probiotic had no significant effect on fecal score (Table 5; *p* > 0.05).

**Table 3:** Effects of dietary complex probiotics supplementation on growth performance in weaned piglets.

Item	CON	Probiotics	SEM	*p* value
Body weight, kg				
Initial	8.53	8.59	0.077	0.619
Day 14	11.12	11.89	0.240	0.035
Day 28	18.55	19.92	0.238	< 0.001
ADFI, g/day				
Day 0–14	312.2	331.7	33.45	0.685
Day 15–28	840.2	818.7	55.65	0.788
Day 0–28	552.5	579.7	23.50	0.422
ADG, g/day				
Day 0–14	185.1	235.7	17.77	0.056
Day 15–28	530.6	573.3	27.68	0.287
Day 0–28	357.9	404.6	7.87	< 0.001
F: G				
Day 0–14	1.69	1.41	0.082	0.023
Day 15–28	1.58	1.43	0.064	0.127
Day 0–28	1.55	1.44	0.061	0.226

CON, Control group; SEM, Standard error of means; ADFI, Average daily feed intake; ADG, Average daily gain; and F: G, The ratio of feed to gain.

**Table 4:** Effects of dietary complex probiotics supplementation on apparent total tract digestibility of nutrient in weaned piglets.

Item, %	CON	Probiotics	SEM	*p* value
Day 14				
Dry matter	87.14	86.76	0.357	0.456
Crude protein	83.94	83.90	0.369	0.935
Gross energy	87.37	87.28	0.290	0.830
Day 28				
Dry matter	85.02	85.65	0.250	0.092
Crude protein	83.48	83.90	0.232	0.211
Gross energy	86.59	86.39	0.269	0.605

CON, Control group; SEM, Standard error of means.

**Table 5:** Effects of dietary complex probiotics supplementation on fecal score in weaned piglets.

Item	CON	Probiotics	SEM	*p* value
Day 7	3.41	3.25	0.255	0.649
Day 14	3.17	3.33	0.304	0.702
Day 21	3.25	3.42	0.283	0.681
Day 28	3.58	3.25	0.309	0.453

CON, Control group; SEM, Standard error of means; Fecal score = 1 hard, dry pellet; 2 firm, formed stool; 3 soft, moist stool that retains shape; 4 soft, unformed stool that assumes the shape of the container; and 5 watery liquid that can be poured.

### 3.2 Hematological profiles

The addition of complex probiotic to the diet resulted in a significant reduction in blood glucose concentration compared to CON (Table 6; *p* < 0.05), while the addition of complex probiotic to the diet did not affect the blood concentrations of IgA, IgG, IgM, IL-1*β*, IL-6, IL-10, albumin, total cholesterol, and total protein (*p* > 0.05).

**Table 6:** Effects of dietary complex probiotics supplementation on hematological profiles in weaned piglets.

Item	CON	Probiotics	SEM	*p* value
IgA, mg/mL	0.97	1.04	0.180	0.971
IgG, mg/mL	7.50	7.36	0.231	0.667
IgM, mg/mL	1.09	0.96	0.142	0.520
IL-1β, pg./mL	27.59	28.30	0.455	0.283
IL-6, pg./mL	86.71	88.64	1.285	0.311
IL-10, pg./mL	31.30	29.79	0.775	0.181
Albumin, g/L	29.83	30.12	0.714	0.872
Total cholesterol, mmol/L	2.98	2.82	0.176	0.523
Glucose, mmol/L	6.68	5.61	0.342	0.037
Total protein, g/L	51.42	50.69	1.279	0.692

CON, Control group; SEM, Standard error of means; IgA, Immunoglobulin A; IgG, Immunoglobulin G; IgM, Immunoglobulin M; IL-1β, Interleukin-1β; IL-6, Interleukin-6; and IL-10, Interleukin-10.

### 3.3 Intestinal morphology and fecal microbiota

**Figure 1:**
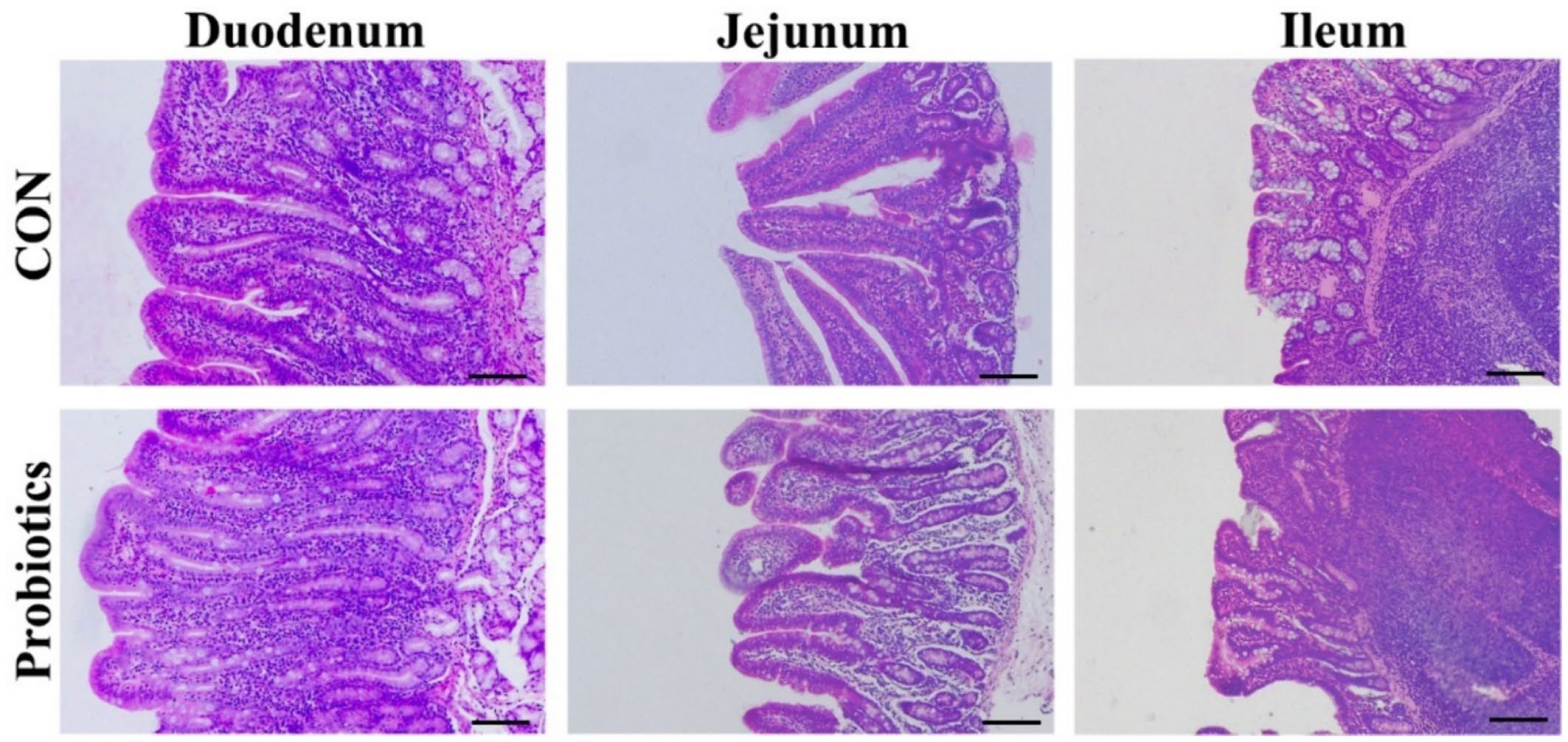
The morphology of duodenum, jejunum, and ileum in weaned piglets fed with basal diets supplemented with complex probiotics (Scale bar: 100 μm).

Table 7 and Figure 1 showed that the dietary complex probiotic addition significantly increased the VH and VH: CD of duodenum and ileum in weaned piglets, compared to CON (*p* < 0.05). However, the addition of complex probiotic to the diet had no significant effect on the morphology of the jejunum in piglets (*p* > 0.05). In addition, we did not find differences in fecal microbiota (Table 8, *p* > 0.05).

**Table 7:** Effects of dietary complex probiotics supplementation on intestinal morphology in weaned piglets.

Item	CON	Probiotics	SEM	*p* value
Duodenum				
VH, um	324.9	428.3	5.523	< 0.001
CD, um	278.3	275.3	5.052	0.681
VH: CD	1.17	1.56	0.031	< 0.001
Jejunum				
VH, um	407.7	409.8	4.192	0.724
CD, um	296.3	296.5	5.240	0.972
VH: CD	1.38	1.39	0.025	0.891
Ileum				
VH, um	275.4	311.4	6.446	< 0.001
CD, um	196.6	188.4	5.297	0.286
VH: CD	1.41	1.67	0.050	0.001

CON, Control group; SEM, Standard error of means; VH, Villus height; CD, Crypt depth; and VH: CD, Villus height: crypt depth.

**Table 8:** Effects of dietary complex probiotics supplementation on fecal microbiota in weaned piglets.

Item, Lg (copies/g)	CON	Probiotics	SEM	*p* value
*Prevotella*	9.70	10.09	0.326	0.405
*Bacteroides*	11.30	10.79	0.359	0.320
*Bifidobacterium*	12.20	10.82	0.502	0.064
*Escherichia coli*	9.48	9.09	0.477	0.570
*Lactobacillus*	7.87	8.10	0.737	0.827
*Bacillus*	6.49	6.76	0.432	0.660

CON, Control group; SEM, Standard error of means.

### 3.4 Fecal SCFAs and fecal gas emissions

Concentrations of acetate, propionate, butyrate, and total SCFAs in feces were significantly higher in the complex probiotic group than in CON (Table 9; *p* < 0.05). Furthermore, the dietary inclusion of complex probiotic significantly decreased the concentrations of methyl mercaptans, acetic acid, and CO_2_ in the feces of piglets on day 28 (Table 10; *p* < 0.05). However, the addition of complex probiotic did not have a significant effect on the concentrations of fecal microbiota (Table 8), isobutyrate, isovalerate, valerate, NH_3_, and H_2_S (*p* > 0.05).

**Table 9:** Effects of dietary complex probiotics supplementation on fecal SCFAs in weaned piglets.

Item, mg/g dry face weight	CON	Probiotics	SEM	*p* value
Acetate	2.62	4.31	0.232	< 0.001
Propionate	1.67	2.56	0.270	0.029
Isobutyrate	0.54	0.52	0.080	0.838
Butyrate	0.39	1.13	0.147	0.002
Isovalerate	0.15	0.23	0.027	0.066
Valerate	0.67	0.68	0.087	0.936
Total SCFAs	6.05	9.42	0.369	< 0.001

CON, Control group; SEM, Standard error of means; SCFAs, Short-chain fatty acids.

**Table 10:** Effects of dietary complex probiotics supplementation on fecal gas emissions in weaned piglets.

Item, ppm	CON	Probiotics	SEM	*p* value
Day 28				
Methyl mercaptans	4.77	3.85	0.252	0.017
NH_3_	3.79	3.35	0.282	0.282
H_2_S	2.30	2.36	0.270	0.871
Acetic acid	8.26	5.65	0.317	<0.001
CO_2_	9,919	8,742	386.5	0.043

CON, Control group; SEM, Standard error of means.

## 4 Discussion

Weaning stress in piglets can have negative economic effects (1, 2). It is a straightforward approach to evaluate economic effects through growth performance. A variety of factors can influence growth performance, feed nutrients, management practices, and external stimuli (30–34). The study results indicated that adding complex probiotic to piglets’ diets effectively increased ADG and decreased F: G during days 0–14. However, there was no effect on ADG and F: G during days 15–28. Throughout the trial, there was no significant difference in ADFI between the two treatment groups. The present study compared the effect of adding four different probiotics mixture to the diet. Previous study has shown that the addition of probiotics either alone or in combination had no impact on feed intake and F: G. Furthermore, the enhancement of the growth performance of piglets by the dietary addition of probiotics was primarily achieved by an increase in the daily weight (17). However, the results of this study showed that the addition of complex probiotic alleviated the negative effects of weaning stress by reducing F: G (1.69–1.41) and enhancing ADG (185.1–235.7 g) in the early stage of weaning (days 0–14). Although ADG (530.6–573.3 g) and F: G (1.58–1.43) were changed to a certain extent in the second half of the piglet’s life (days 15–28), but the effect was not significant. However, in a study where *Bacillus licheniformis* was added alone, the results indicated that there was no significant effect on the ADG of piglets during the first 14 days, but there was a significant increase in ADG on days 14–28 (35). Another study showed that the addition of *Saccharomyces cerevisiae* to diets also observed significant differences in body weight over time (15). The above results are not consistent with the results of the present study. In conclusion, the aforementioned results indicated that the phase of interest when studying the dietary complex probiotics addition may be 2 weeks after weaning. Previous research has indicated that weaning stress can cause diarrhea and decrease the height of the intestinal villi (3, 4). This study found that there was no significant difference in fecal scores between the two treatment groups. The mean value of the scores ranged from 3 to 4 (moist stool that retains shape). This study did not find any evidence of severe diarrhea. The same result was observed in the study of *Bacillus licheniformis* as a sole component of the diet.

Nutrients, including glucose, amino acids, glycerol, and fatty acids, are primarily absorbed in the small intestine. The height of the small intestinal villi affects the uptake and absorption of nutrients to some extent. Although this study found that the addition of complex probiotic increased the VH of the duodenum and ileum, there was no significant effect on the apparent total tract digestibility of the nutrients. The result suggested that the growth performance of the complex probiotic group may be improved due to the different fermentation capacities of the hindgut microorganisms.

The mammalian gut harbors a considerable and heterogeneous microbial community. The host and gut microbes are mutually dependent, coordinating and constraining each other in order to achieve homeostasis within the organism (36, 37). SCFAs are the end-products of intestinal bacterial fermentation with various metabolic characteristics. They can provide energy for the intestinal flora and the host’s epithelial cells, maintain intestinal acid–base balance, inhibit the growth of harmful pathogens, and regulate the host’s intestinal immune system so as to alleviate the inflammatory response (38). In the study, we analyzed the fecal microbiota content and found no significant difference between the two treatment groups. Then, by measuring fecal SCFAs, we found that concentrations of acetate, propionate, butyrate, and total SCFAs in feces were significantly higher in the complex probiotic group than in CON. The results indicated that the fermentation capacity of gut microorganisms was higher in the complex probiotic group. Nevertheless, the observed differences were not statistically significant in terms of the measured gut microbial content. Consequently, the use of alternative methods may be required to determine a more comprehensive range of gut microbial species and content. Butyric acid and acetate can be absorbed by the hindgut and provide a significant amount of energy for the body (39). This could be the primary reason why adding complex probiotic to the diet enhanced growth performance. Additionally, we analyzed the blood profile of the piglets. IL-6 and IL-10 are associated with inflammatory responses (40–42). IgA, IgG, and IgM are associated with immune function (43–45). However, this study did not find any differences in the aforementioned factors between the two treatment groups. However, a significant reduction in blood glucose levels was found in the probiotic complex group. Previous studies have shown that higher levels of SCFAs can significantly reduce plasma glucose levels (46, 47). Its primary function is to regulate blood glucose levels through the hepatic AMP-activated protein kinase pathway and to affect blood glucose levels by increasing intestinal satiety hormones (47). The results indicated that the probiotic complex group changed the composition of fecal SCFAs, reduced blood glucose levels, and improved growth performance.

With the intensification of the pig industry, the impact on emissions of environmental pollution and animal health becomes increasingly significant. Studies have shown that emissions from large farms can spread up to 5 km in the vicinity (48–50). Harmful gases such as methyl mercaptans, acetic acid, H_2_S, and NH_3_ can cause respiratory damage in pigs and reduce growth performance (51). The results of this study showed that the dietary complex probiotic supplementation significantly decreased the concentrations of methyl mercaptans, acetic acid, and CO_2_ in the feces of piglets. In conclusion, the addition of dietary complex probiotic can effectively reduce the level of harmful gases in the pig house.

## 5 Conclusion

Dietary supplementation with 0.2% probiotics improved gut health by increasing duodenal and ileal VH, and increased final BW by increasing ADG.

## Data availability statement

The raw data supporting the conclusions of this article will be made available by the authors, without undue reservation.

## Ethics statement

The animal studies were approved by Committee of Southwest University of Science and Technology. The studies were conducted in accordance with the local legislation and institutional requirements. Written informed consent was obtained from the owners for the participation of their animals in this study.

## Author contributions

JZ: Conceptualization, Data curation, Writing – original draft, Writing – review & editing. ZX: Data curation, Writing – original draft. MZ: Data curation, Writing – review & editing. WT: Software, Writing – review & editing. HD: Writing – review & editing. HY: Conceptualization, Data curation, Methodology, Writing – original draft, Writing – review & editing.

## References

[1] Lallès J-P Bosi P Smidt H Stokes CR. Weaning — a challenge to gut physiologists. Livest Sci. (2007) 108:82–93. doi: 10.1016/j.livsci.2007.01.091

[2] Liao SF Nyachoti M. Using probiotics to improve swine gut health and nutrient utilization. Anim Nutr. (2017) 3:331–43. doi: 10.1016/j.aninu.2017.06.007, PMID: 29767089 PMC5941265

[3] O’Loughlin A McGee M Waters SM Doyle S Earley B. Examination of the bovine leukocyte environment using immunogenetic biomarkers to assess immunocompetence following exposure to weaning stress. BMC Vet Res. (2011) 7:45. doi: 10.1186/1746-6148-7-45, PMID: 21834971 PMC3177877

[4] Boudry G Péron V Le Huërou-Luron I Lallès JP Sève B. Weaning induces both transient and long-lasting modifications of absorptive, secretory, and barrier properties of piglet intestine. J Nutr. (2004) 134:2256–62. doi: 10.1093/jn/134.9.2256, PMID: 15333713

[5] Arif M Iram A Bhutta MAK Naiel MAE Abd El-Hack ME Othman SI. The biodegradation role of *Saccharomyces cerevisiae* against harmful effects of mycotoxin contaminated diets on broiler performance, immunity status, and carcass characteristics. Animals. (2020) 10:238. doi: 10.3390/ani10020238, PMID: 32028628 PMC7070355

[6] Alagawany M Abd El-Hack ME Farag MR Sachan S Karthik K Dhama K. The use of probiotics as eco-friendly alternatives for antibiotics in poultry nutrition. Environ Sci Pollut Res. (2018) 25:10611–8. doi: 10.1007/s11356-018-1687-x, PMID: 29532377

[7] Hori T Matsuda K Oishi K. Probiotics: a dietary factor to modulate the gut microbiome, host immune system, and gut-brain interaction. Microorganisms. (2020) 8:1401. doi: 10.3390/microorganisms8091401, PMID: 32933067 PMC7563712

[8] Meng QW Yan L Ao X Zhou TX Wang JP Lee JH. Influence of probiotics in different energy and nutrient density diets on growth performance, nutrient digestibility, meat quality, and blood characteristics in growing-finishing pigs. J Anim Sci. (2010) 88:3320–6. doi: 10.2527/jas.2009-2308, PMID: 20562363

[9] Xuan ZN Kim JD Heo KN Jung HJ Lee JH Han YK. Study on the development of a probiotics complex for weaned pigs. Asian Australas J Anim Sci. (2001) 14:1425–8. doi: 10.5713/ajas.2001.1425

[10] Giang HH Viet TQ Ogle B Lindberg JE. Effects of supplementation of probiotics on the performance, nutrient digestibility and Faecal microflora in growing-finishing pigs. Asian Australas J Anim Sci. (2011) 24:655–61. doi: 10.5713/ajas.2011.10238

[11] Roselli M Pieper R Rogel-Gaillard C de Vries H Bailey M Smidt H. Immunomodulating effects of probiotics for microbiota modulation, gut health and disease resistance in pigs. Anim Feed Sci Technol. (2017) 233:104–19. doi: 10.1016/j.anifeedsci.2017.07.011

[12] Dong X Zhang N Zhou M Tu Y Deng K Diao Q. Effects of dietary probiotics on growth performance, faecal microbiota and serum profiles in weaned piglets. Anim Prod Sci. (2013) 54:616–21. doi: 10.1071/AN12372

[13] Wang J Li S Tang W Diao H Zhang H Yan H. Dietary complex probiotic supplementation changed the composition of intestinal short-chain fatty acids and improved the average daily gain of growing pigs. Vet Sci. (2023) 10:79. doi: 10.3390/vetsci10020079, PMID: 36851383 PMC9965097

[14] Vieira AM Sessin AP Soratto TAT Pires PGDS Cardinal KM Wagner G. Effect of functional oils or probiotics on performance and microbiota profile of newly weaned piglets. Sci Rep. (2021) 11:19457. doi: 10.1038/s41598-021-98549-w, PMID: 34593866 PMC8484476

[15] Kiros TG Luise D Derakhshani H Petri R Trevisi P D’Inca R. Effect of live yeast *Saccharomyces cerevisiae* supplementation on the performance and cecum microbial profile of suckling piglets. PLoS One. (2019) 14:e0219557. doi: 10.1371/journal.pone.0219557, PMID: 31329605 PMC6645501

[16] Deng B Wu J Li X Zhang C Men X Xu Z. Effects of *Bacillus subtilis* on growth performance, serum parameters, digestive enzyme, intestinal morphology, and colonic microbiota in piglets. AMB Express. (2020) 10:212. doi: 10.1186/s13568-020-01150-z, PMID: 33263814 PMC7710768

[17] Qiao J Li H Wang Z Wang W. Effects of *Lactobacillus acidophilus* dietary supplementation on the performance, intestinal barrier function, rectal microflora and serum immune function in weaned piglets challenged with *Escherichia coli* lipopolysaccharide. Antonie Van Leeuwenhoek. (2015) 107:883–91. doi: 10.1007/s10482-015-0380-z, PMID: 25577203

[18] Yu X Cui Z Qin S Zhang R Wu Y Liu J. Effects of *Bacillus licheniformis* on growth performance, diarrhea incidence, antioxidant capacity, immune function, and fecal microflora in weaned piglets. Animals. (2022) 12:1609. doi: 10.3390/ani12131609, PMID: 35804509 PMC9264952

[19] Weichselbaum E. Probiotics and health: a review of the evidence. Nutr Bull. (2009) 34:340–73. doi: 10.1111/j.1467-3010.2009.01782.x

[20] National Research Council. Nutrient Requirements of Swin. 11th ed. Washington, DC: The National Academies Press (2012). 420 p.

[21] AOAC (2007). Official methods of analysis. 18th ed. Arlington (VA): Association of Official Analytical Chemists. Available at: https://www.docin.com/p-933447075.html (Accessed July 31, 2022).

[22] Liu J Pu Y-Y Xie Q Wang J-K Liu J-X. Pectin induces an in vitro rumen microbial population shift attributed to the Pectinolytic Treponema group. Curr Microbiol. (2015) 70:67–74. doi: 10.1007/s00284-014-0672-y, PMID: 25178631

[23] Layton A McKay L Williams D Garrett V Gentry R Sayler G. Development of Bacteroides 16S rRNA gene Taq man-based real-time PCR assays for estimation of Total, human, and bovine fecal pollution in water. Appl Environ Microbiol. (2006) 72:4214–24. doi: 10.1128/AEM.01036-05, PMID: 16751534 PMC1489674

[24] Bergström A Licht TR Wilcks A Andersen JB Schmidt LR Grønlund HA. Introducing GUt low-density Array (GULDA) – a validated approach for qPCR-based intestinal microbial community analysis. FEMS Microbiol Lett. (2012) 337:38–47. doi: 10.1111/1574-6968.12004, PMID: 22967145

[25] Walker AW Ince J Duncan SH Webster LM Holtrop G Ze X. Dominant and diet-responsive groups of bacteria within the human colonic microbiota. ISME J. (2011) 5:220–30. doi: 10.1038/ismej.2010.118, PMID: 20686513 PMC3105703

[26] Huijsdens XW Linskens RK Mak M Meuwissen SGM Vandenbroucke-Grauls CMJE Savelkoul PHM. Quantification of Bacteria adherent to gastrointestinal mucosa by real-time PCR. J Clin Microbiol. (2002) 40:4423–7. doi: 10.1128/JCM.40.12.4423-4427.2002, PMID: 12454130 PMC154607

[27] Khafipour E Li S Plaizier JC Krause DO. Rumen microbiome composition determined using two nutritional models of subacute ruminal acidosis. Appl Environ Microbiol. (2009) 75:7115–24. doi: 10.1128/AEM.00739-09, PMID: 19783747 PMC2786511

[28] Fernández-No IC Guarddon M Böhme K Cepeda A Calo-Mata P Barros-Velázquez J. Detection and quantification of spoilage and pathogenic *Bacillus cereus*, Bacillus subtilis and *Bacillus licheniformis* by real-time PCR. Food Microbiol. (2011) 28:605–10. doi: 10.1016/j.fm.2010.10.014, PMID: 21356471

[29] Wang M Wang L Tan X Wang L Xiong X Wang Y. The developmental changes in intestinal epithelial cell proliferation, differentiation, and shedding in weaning piglets. Anim Nutr. (2022) 9:214–22. doi: 10.1016/j.aninu.2021.11.006, PMID: 35600553 PMC9092860

[30] Johnson JS Aardsma MA Duttlinger AW Kpodo KR. Early life thermal stress: impact on future thermotolerance, stress response, behavior, and intestinal morphology in piglets exposed to a heat stress challenge during simulated transport 1. J Anim Sci. (2018) 96:1640–53. doi: 10.1093/jas/sky107, PMID: 29635346 PMC6140855

[31] Cao SC Gong J Wang J Yan HL Zhang HF Liu JB. The impact of the interaction between dietary total phosphorus level and efficacy of phytase on the performance of growing-finishing pigs. Anim Feed Sci Technol. (2023) 298:115605. doi: 10.1016/j.anifeedsci.2023.115605

[32] Zhai H Adeola O Liu J. Phosphorus nutrition of growing pigs. Anim Nutr. (2022) 9:127–37. doi: 10.1016/j.aninu.2022.01.010, PMID: 35573097 PMC9079227

[33] Cao S Wang J Zhao J Li S Tang W Diao H. Dietary soybean oligosaccharides addition increases growth performance and reduces lipid deposition by altering fecal short-chain fatty acids composition in growing pigs. Animals. (2023) 13:3648. doi: 10.3390/ani13233648, PMID: 38066999 PMC10705736

[34] Cao S Yan H Tang W Zhang H Liu J. Effects of dietary coenzyme Q10 supplementation during gestation on the embryonic survival and reproductive performance of high-parity sows. J Anim Sci Biotechnol. (2023) 14:75. doi: 10.1186/s40104-023-00879-4, PMID: 37264441 PMC10236690

[35] Cao G Yang S Wang H Zhang R Wu Y Liu J. Effects of *Bacillus licheniformis* on the growth performance, antioxidant capacity, Ileal morphology, intestinal short chain fatty acids, and colonic microflora in piglets challenged with lipopolysaccharide. Animals. (2023) 13:2172. doi: 10.3390/ani13132172, PMID: 37443970 PMC10340043

[36] Felizardo RJF Watanabe IKM Dardi P Rossoni LV Câmara NOS. The interplay among gut microbiota, hypertension and kidney diseases: the role of short-chain fatty acids. Pharmacol Res. (2019) 141:366–77. doi: 10.1016/j.phrs.2019.01.019, PMID: 30639376

[37] Qiao Y Sun J Xia S Tang X Shi Y Le G. Effects of resveratrol on gut microbiota and fat storage in a mouse model with high-fat-induced obesity. Food Funct. (2014) 5:1241–9. doi: 10.1039/C3FO60630A, PMID: 24722352

[38] Sadler R Cramer JV Heindl S Kostidis S Betz D Zuurbier KR. Short-chain fatty acids improve Poststroke recovery via immunological mechanisms. J Neurosci. (2020) 40:1162–73. doi: 10.1523/JNEUROSCI.1359-19.2019, PMID: 31889008 PMC6989004

[39] Binder HJ. Role of colonic short-chain fatty acid transport in diarrhea. Annu Rev Physiol. (2010) 72:297–313. doi: 10.1146/annurev-physiol-021909-135817, PMID: 20148677

[40] Degré M. Interferons and other cytokines in bacterial infections. J Interf Cytokine Res. (1996) 16:417–26. doi: 10.1089/jir.1996.16.417 8807494

[41] Conti P Kempuraj D Kandere K Gioacchino MD Barbacane RC Castellani ML. IL-10, an inflammatory/inhibitory cytokine, but not always. Immunol Lett. (2003) 86:123–9. doi: 10.1016/S0165-2478(03)00002-6, PMID: 12644313

[42] Opal SM DePalo VA. Anti-inflammatory cytokines. Chest. (2000) 117:1162–72. doi: 10.1378/chest.117.4.1162 10767254

[43] Chodirker WB Tomasi TB. Gamma-globulins: quantitative relationships in human serum and nonvascular fluids. Science. (1963) 142:1080–1. doi: 10.1126/science.142.3595.1080, PMID: 14068229

[44] Vidarsson G Dekkers G Rispens T. IgG subclasses and Allotypes: from structure to effector functions. Front Immunol. (2014) 5:520. doi: 10.3389/fimmu.2014.00520 25368619 PMC4202688

[45] Keyt BA Baliga R Sinclair AM Carroll SF Peterson MS. Structure, function, and therapeutic use of IgM antibodies. Antibodies. (2020) 9:53. doi: 10.3390/antib9040053 33066119 PMC7709107

[46] Fushimi T Suruga K Oshima Y Fukiharu M Tsukamoto Y Goda T. Dietary acetic acid reduces serum cholesterol and triacylglycerols in rats fed a cholesterol-rich diet. Br J Nutr. (2006) 95:916–24. doi: 10.1079/BJN20061740, PMID: 16611381

[47] Sakakibara S Yamauchi T Oshima Y Tsukamoto Y Kadowaki T. Acetic acid activates hepatic AMPK and reduces hyperglycemia in diabetic KK-A (y) mice. Biochem Biophys Res Commun. (2006) 344:597–604. doi: 10.1016/j.bbrc.2006.03.176, PMID: 16630552

[48] Giraldi-Díaz MR Castillo-González E De Medina-Salas L Velásquez-De la Cruz R Huerta-Silva HD. Environmental impacts associated with intensive production in pig farms in Mexico through life cycle assessment. Sustain For. (2021) 13:11248. doi: 10.3390/su132011248

[49] Calvet S Hunt J Misselbrook TH. Low frequency aeration of pig slurry affects slurry characteristics and emissions of greenhouse gases and ammonia. Biosyst Eng. (2017) 159:121–32. doi: 10.1016/j.biosystemseng.2017.04.011, PMID: 28713224 PMC5492791

[50] Pirlo G Carè S Casa GD Marchetti R Ponzoni G Faeti V. Environmental impact of heavy pig production in a sample of Italian farms. A cradle to farm-gate analysis. Sci Total Environ. (2016) 565:576–85. doi: 10.1016/j.scitotenv.2016.04.174, PMID: 27203518

[51] Curtis SE Anderson CR Simon J Jensen AH Day DL Kelley KW. Effects of aerial Ammonia, hydrogen sulfide and swine-house dust on rate of gain and respiratory-tract structure in swine. J Anim Sci. (1975) 41:735–9. doi: 10.2527/jas1975.413735x, PMID: 1158807

